# Development of a Granule Growth Regime Map for Twin Screw Wet Granulation Process via Data Imputation Techniques

**DOI:** 10.3390/pharmaceutics14102211

**Published:** 2022-10-17

**Authors:** Lalith Kotamarthy, Chaitanya Sampat, Rohit Ramachandran

**Affiliations:** Department of Chemical and Biochemical Engineering, Rutgers, The State University of New Jersey, Piscataway, NJ 08854, USA

**Keywords:** twin screw granulation, regime map, data imputation, granule growth, granulation regimes, granulation mechanisms

## Abstract

Twin screw granulation (TSG) is a continuous wet granulation technique that is used widely across different solid manufacturing industries. The TSG has been recognized to have numerous advantages due to its modular design and continuous manufacturing capabilities, including processing a wide range of formulations. However, it is still not widely employed at the commercial scale because of the lack of holistic understanding of the process. This study addresses that problem via. the mechanistic development of a regime map that considers the complex interactions between process, material, and design parameters, which together affect the final granule quality. The advantage of this regime map is that it describes a more widely applicable quantitative technique that can predict the granule growth behavior in a TSG. To develop a robust regime map, a database of various input parameters along with the resultant final granule quality attributes was created using previously published literature experiments. Missing data for several quality attributes was imputed using various data completion techniques while maintaining physical significance. Mechanistically relevant non-dimensional X and Y axis that quantify the physical phenomena occurring during the granulation were developed to improve the applicability and predictability of the regime map. The developed regime map was studied based on process outcomes and granule quality attributes to identify and create regime boundaries for different granule growth regimes. In doing so breakage-dominant growth was incorporated into the regime map, which is very important for TSG. The developed regime map was able to accurately explain the granule growth regimes for more than 90% of the studied experimental points. These experimental were generated at vastly different material, design, and process parameters across various studies in the literature, this further increases the confidence in the developed regime map.

## 1. Introduction

Wet granulation is a process of converting fine primary powder particles to larger agglomerates using a liquid binder and also served to improve the physical properties of the material, making it easier to handle and process in downstream manufacturing. Wet granulation is commonly used to manufacture food materials, detergents, minerals, catalysts and pharmaceuticals [1]. Traditionally, wet granulation is performed in a batch mode. the United States Food and Drug Administration (US-FDA) has encouraged the pharmaceutical industry to adopt a more flexible, agile, and efficient mode of manufacturing using a continuous mode. The adoption of continuous manufacturing in the solid oral dosage pharmaceutical industry, involves the systematic integration of several unit operations, process knowledge and automation tools With the added push of concepts such as Quality by Design (QbD) and Process Analytical Technology (PAT) [2], continuous manufacturing became very popular as it has several benefits over batch production [3,4].

Continuous wet granulation can handle a higher throughput of material [5], has predictable product quality and has less product development time [1]. Twin screw granulators (TSGs) are one of the most used continuous granulation equipment. A TSG consists of two co-rotating screws enclosed in a barrel. These co-rotating screws help convey the material along the length of the granulator while also imparting shear onto the flowing powders. This applied mechanical energy aids liquid distribution and mixing in the different zones in the granulator [6,7]. The screws of the TSG are modular due to the usage of smaller individual screw elements [1]. Three commonly used screw elements are (i) conveying elements which help in transport of material [5,8,9], (ii) kneading elements which help in both liquid mixing and imparting shear on to the powders [8,10], and (iii) comb-mixer elements which aid in liquid distribution [11,12,13,14]. These elements can be arranged in different ways on the screws and therefore, there are a large number of possible screw configurations. Similarly, there exists several sizes and geometry of the TSGs currently being produced by manufacturers. The combination of the process parameters, material properties, screw configurations and the various TSG sizes leads to a large number of permutations which can make developing a predicitive process model for a TSG a challenging task [15].

Wet granulation is governed by three important mechanisms namely (i) Wetting and Nucleation (ii) Consolidation and Growth (iii) Attrition and Breakage [6]. Understanding these mechanisms is very important as they determine the critical quality attributes of granules such as particle size distribution (PSD), content uniformity (CU), granule micro-structure, etc. Thus, it is important to develop apriori techniques that can predict their effect on granule growth.

To understand the effect of various granulation process and geometry variables on the granule CQAs, a regime map theory can be applied [16]. A regime map helps correlate the granule growth characteristics with the input process variables, geometry used and the materials used for granulation [17]. It also helps in determining the design space of experiments with fewer initial experiments [18]. The development of a regime map requires the understanding of the interactions between process parameters, material properties and geometry. Several researchers have investigated the effects of formulation [5,19], screw configuration [20,21,22] and process variables [23] using different granulator sizes and limited scales of operation. These studies analyzed the individual effects or an interaction of a few effects on outputs but understanding correlations between a large variety of variables still remains unexplored.

Unlike in a batch high shear or fluid bed granulation process, in a twin-screw granulation process, the regimes are physically separated along the length of the screws [6]. Moreover, due to the lack of space available for granules to grow in a TSG, there is an increase in the stress applied per unit time on the granules. These differences necessitate the development of a modified regime maps for the twin-screw granulation process. One of first regime maps for the TSG was developed by Dhenge et al. [5], by drawing similarities to the regime map development by previously performed by Iveson et al. [17]. However, it has adapted this to a continuous process by assuming that the different granulation mechanisms occurred along the length of the barrel and no re-circulation was present. Furthermore, Dhenge et al. [5] defined regimes differently: under-wetted(dry), nucleation, crumb, granules and over-wetted/paste. The *y*-axis chosen consisted of the deformation value (β) which is an indication of the system deformability. This *y*-axis replaced the Stokes deformation number ($St_{De}$) used in the study by Iveson and Litster [24]. With a constant screw configuration, Dhenge et al. [5] studied the effect of binder liquid viscosity and surface tension on the granule properties by varying the *L*/*S* ratios. It was observed that in all cases the largest granules were obtained for the formulation with the highest viscosity. All the experiments performed in the study by Dhenge et al. [5] were performed in the crumb and granule regime. As an extension to the previous study, Dhenge et al. [19] used a conveying element only screw configuration to study the effects of varying liquid viscosity and powder feed rate on granule properties. The shear forces within the TSG system were low due to the use of only conveying elements. This resulted in granule formation occurring in the nucleation regime. It was also observed that with increase in viscosity of the binder the fraction of fines and smaller granules increased. This was attributed to the lack of sufficient shear forces. Granules formed with water had a higher deformation value than the granules formed using more viscous binders, leading to the aforementioned observations. They also concluded that the conveying elements did not aid in homogeneous distribution of the liquid binder. The two developed regime maps were screw configuration specific and the boundaries between different granule regimes were not analyzed mechanistically rather only made a qualitative distinction based on the experimental result.

Tu et al. [25] developed a regime map for a TSG that studied the effect of *L*/*S* ratio, screw speed and screw configuration. Tu et al. [25] observed that an increase in the screw speed led to a decrease in the fill level of the TSG which in turn led to a decrease in the frictional force and torque values for the system. They divided the granule regimes as: granulation, extrudate and blocked based on the screw configuration. The use of scale-dependent material also made the developed regime map not general in nature and the boundaries between the regime were not well-defined. Kumar et al. [15] developed a process map based on a full factorial design of experiment (DoE). Kumar et al. [15] varied various process variables such as powder feed rate, liquid to solid ratio, screw configuration and screw speed. They observed that larger granules were obtained at lower *L*/*S* ratio especially when two kneading zones were used in the screw configuration instead of one. They also observed that the increase in energy input decreased the median granule diameter. They recognized that the developed process map had limited applicability and that more studies with a wide variety of formulations, equipment and process conditions would be required in order to create a more general map. Pohl et al. [26] in accordance with observations of Kumar et al. [15] suggested that the barrel filling degree and the residence time of the material inside the TSG are important factors and their effects on the granules and granule properties need to be investigated further for the development of the next regime map. Thus, there still remains a need for the development of a regime map for a TSG that can take in account different equipment, screw configurations, material properties and process variables [27].

### Objectives

In the presented study, we address the lack of a more general regime for the TSG process using historical experimental data. Here, we develop a regime map that has a larger general applicability compared to previously developed regime maps and aimed to define the boundaries separating the regimes more mechanistically. Previously published experimental data from several researchers was sourced from the literature to create an comprehensive database where several formulations, screw configuration, active pharmaceutical ingredients (APIs), binders, granulator scales, and process parameters were considered. As not all researchers reported all necessary outputs required for the development of the general regime map, the missing data was completed using robust data completion techniques. Where possible, first-principle based equations were used to complete these outputs. In some cases, where such relations were not present several statistical techniques were compared and the outputs with variability close to actual experiments were chosen. Once a complete data set was obtained, mechanistically relevant non-dimensional *x*-axis and *y*-axis were determined in order to obtain the regime map. Based on the extent of granulation, granule density, granule strength and several other process and intermediate variables, the final regimes and the boundaries separating them were determined.

## 2. Background

### 2.1. Data Completion Methods

Recently, Mundozah et al. [28] developed a geometrical model that relates the volumetric flowrate to the forward volumetric conveying rate. However, estimating the cross-sectional area for screw elements is difficult to calculate for different elements. To complete the fill level data for several of sets experiments, a more general fill level correlation was required which took into account geometrical measurements that could be scaled between different TSG sizes. Osorio et al. [29] developed a correlation for fill level which was scalable and could be used across several TSG sizes. The fill level developed by Osorio et al. [29] is shown in Equation (1). This correlation represents a complex relation between the mass flow rate and screw geometry.

(1) $$\begin{array}{c} \phi =\frac{1}{F_{1}F_{2}F_{3}}PFN \end{array}$$

where, PFN is the powder feed number which is the ratio of how quickly the powder is fed into the TSG barrel to the screw turnover volume. $F_{1},F_{2}\;\&\;F_{3}$ are geometric ratios that describe the geometry of individual screws and the screw configuration. PFN is defined as shown in Equation (2).

(2) $$\begin{array}{c} PFN=\frac{\dot{m_{p}}}{\rho_{b}\omega D^{3}} \end{array}$$

where, $\dot{m_{p}}$ is the mass flow rate of the powder, $\rho_{b}$ represents the bulk density of the powder, ω is the angular velocity of the shaft and *D* is the barrel diameter. The geometric ratios $F_{1},F_{2}\;\&\;F_{3}$ are defined as follows:

(3) $$\begin{array}{c} F_{1}=\frac{A_{elem}}{D^{2}} \end{array}$$

(4) $$\begin{array}{c} F_{2}=\frac{L_{elem}}{D} \end{array}$$

(5) $$\begin{array}{c} F_{3}=\frac{2\pi v_{p}}{\omega L_{elem}} \end{array}$$

In Equation (3), $A_{elem}$ is the free the cross-sectional area of the element perpendicular to the barrel length, $L_{elem}$ is length of the element. $F_{2}$ in Equation (4) is the length to diameter ratio. $v_{p}$ in Equation (5) is the net forward velocity of the powder.

### 2.2. Granulation Regime Maps

A granulation regime map is a technique that is represented as a two-dimensional plot explaining the effect of various process, equipment, and material properties on the granulation rate mechanism considered in the regime map [17,24,30]. Typically, a granulation rate regime map is divided into different regions based on the location on the 2-D plot. These individual regions explain the nature of the granulation rate mechanism occurring at a particular point, subject to conditions specified by the *x* and *y*-axis at that point.

To develop a granulation growth regime map, it is important to understand the fundamentals of the granule growth mechanism. According to common wet-granulation theory, the growth of two surface-wet-colliding granules occurs via coalescence. During this process of coalescence, the granular structure is consolidated (due to the shear applied on the granules by the system) and the trapped liquid is squeezed to the surface of the newly coalesced granule to promote further growth [31]. This is especially true for high agitation intensity processes such as high shear granulation and twin-screw granulation [32]. For a collision between two surface wet granules to result in a successful coalescence, the collision kinetic energy should be dissipated in the process. This implies that the individual colliding granules have a zero coefficient of restitution value with respect to each other post-collision. Depending on the availability of the available surface liquid between the colliding granules, two scenarios satisfy this condition: (i) If the quantity of surface liquid is sufficiently high such that the collision kinetic energy can be dissipated by the viscous binder layer between the two granules then coalescence will occur, this is known as Type 1 coalescence. (ii) If the surface liquid available is not sufficient for type 1 coalescence, then a successful coalescence occurs if the deformation between the granules on collision is adequately high to sufficiently increase the contact surface area between the two colliding granules. The increased surface area between colliding granules ensures a strong bond formation, this is known as Type 2 coalescence. In this case, the collision kinetic energy is dissipated as the granule deformation energy [24,33].

Granule deformability, which is an important property for Type 2 coalescence is generally incorporated into the *y*-axis of the granulation regime map. Granule deformation is a complex function of the material properties and process parameters employed. Extensive studies have been performed to understand and quantify these effects on granule deformation dynamics [33,34]. For example, Liu et al. [33] quantified the criterion for type 1 and type 2 coalescence by assuming the granules to be elastic-plastic solid and the liquid to be an incompressible Newtonian liquid. By neglecting liquid capillary forces they found that if the condition represented by Equation (6) is satisfied then type 1 coalescence occurs and if the condition represented by Equation (7) is satisfied then type 2 coalescence occurs.

(6) $$\begin{array}{c} St_{v}<\left(\frac{h_{0}}{h_{a}}\right) \end{array}$$

(7) $$\begin{array}{c} {\left(\frac{Y_{d}}{E^{*}}\right)}^{0.5}St_{def}^{-9/8}<\;\frac{0.172}{St_{v}}{\left(\frac{\tilde{D}}{h_{0}}\right)}^{2}{\left[1-\frac{1}{St_{v}}\ln\left(\frac{h_{0}}{h_{a}}\right)\right]}^{1.25}\left(\frac{h_{0}^{2}}{h_{a}^{2}}-1\right) \end{array}$$

Here $St_{v}$, is viscous Stokes number, $h_{0}$ is the liquid layer thickness, $h_{a}$ is the height of granule surface asperities, $Y_{d}$ is the granule dynamic yield stress, $E^{*}$ is the Youngs modulus of the combined granule, $St_{def}$ is the Stokes deformation number and, $\tilde{D}$ is the harmonic mean granule diameter of the two unequal-sized granules involved in the collision.

Based on this theory, $St_{def}$ was chosen as the *y*-axis formula for the batch granulation regime map [17] (Equation (8)). It is defined as the ratio of the externally applied kinetic energy to the energy required for deformation. As the Stokes deformation number increases the value on the left-hand side of Equation (3) decreases, thereby, satisfying the condition for type 2 coalescence.

(8) $$\begin{array}{c} St_{def}=\frac{\rho_{g}U_{c}^{2}}{2Y_{d}} \end{array}$$

Here, $\rho_{g}$ is the granule density, $U_{c}$ is the representative impact velocity and $Y_{d}$ is the granule dynamic yield stress.

Typically, Type 1 coalescence is included in the *x*-axis of the regime map. Iveson et al. [24] postulated that the granule saturation will vary during the granulation as granules consolidate which in turn will affect the Type 1 coalescence. Hence they proposed maximum granule pore saturation as the *x*-axis parameter, which is a measure of available surface liquid content. Maximum granule pore saturation is given by Equation (9).

(9) $$\begin{array}{c} S_{max}=\frac{L/S\;ratio\times \rho_{s}\times \left(1-\epsilon_{min}\right)}{\rho_{l}\times \epsilon_{min}} \end{array}$$

Here, $\rho_{s}$ is the density of the input powder, $\rho_{l}$ is the density of binder liquid, and $\epsilon_{min}$ is defined as the minimum porosity the formulation reaches for that particular set of operating conditions.

## 3. Methods

### 3.1. Data Acquisition

All previous regime maps developed were based on a narrow set of experimental observations performed for that particular study [5,19,24]. To develop a widely applicable regime map it is important to incorporate experimental data that takes into account the wide range of across the entire spectrum of TSG parameter operability. Since, no experiments were performed for this work, it is vital that the experimental data sets collected for the development of the general regime map accounts for a large operating range in terms of process parameters, material properties, different TSG sizes and various screw configurations. The data were collated using previously published experimental data sets as shown in Table 1. A total of 132 data points were collected from previously published 6 data sets. It can be observed from Table 1 that the experimental data collected was representative of the wide parameter ranges that the TSG was operated on.

#### Inputs and Outputs

Granule growth within a granulation process can be inferred from the process outcomes and critical quality attributes (CQAs) of the granules obtained. Some of the process outcomes of the TSG process commonly studied are residence time distribution (RTD), fill level, mixing index, torque inside the system, while granule size distribution (GSD) and granule density/porosity are the commonly studied granule quality attributes [1]. The effect of process parameters such as throughput and RPM on the RTD and mean residence time (MRT) have been studied [21,37]. Lee et al. [37] have also reported that the staggering angle of the kneading element had an effect on the MRT of the system. Several authors have observed that the increase in liquid to solid ratio leads to an increase in the final granule size [20,38]. The fill level (or % fill of barrel) is affected by the specific feed load (SFL) which is the ratio of powder feed rate and screw speed [39]. This fill level in turn influences the final average diameters of the obtained granules [15,40]. The viscosity of the binder used for granulation also seems to affect the growth of the granules and barrel fill level inside a TSG [5,15,19]. Understanding the individual effect of each of the inputs as well the several interactions that affect the granule quality and process outcomes is important in order to obtain the general granule growth regime map. Table 2 lists all the input parameters and outputs collected from each of the sources in order to develop the granule growth regime map for a TSG process. In some literature, outputs had been reported in figures and each figure was processed individually for relevant data. The data from each plot was extracted using WebPlotDigitizer [41]. It is an application developed in HTML5 which helps facilitate extraction of data from various plot types and formats. The tools requires 4 reference points inputs for determining the axis and pixel scaling, through which it is able to extract the date presented in the plots.

### 3.2. Data Imputation Techniques

On further analysis of the collected data, it was realized that several output parameters were missing / incomplete. Out of the 132 points, MRT values were present for only 60 points and torque was only reported for 72 experimental runs. The lowest reported values were for percent barrel fill, where only 42 out of 132 experiments had reported them. If the incomplete data points were to be dropped, there would only be 42 usable points which meant that two thirds of the data points would have to be discarded. Thus, data imputation techniques were necessary in order to have a complete larger data set for improved analysis.

#### 3.2.1. First-Principle Based Data Completion

The barrel fill level for a twin-screw granulator is the ratio of the volume occupied by the powder and the granules to the maximum free volume of the barrel channel [42]. Gorringe et al. [43] proposed a geometrical correlation for predicting the fill level which was related to the mass flow rate and the theoretical maximum capacity of the screws required to convey the powder. The challenge with using this correlation was its dependence on the estimation of volumetric efficiency of the screw used to convey the powder.

In order to estimate fill level using Equation (1) several intermediate values were required. The geometric ratios were calculated using dimensions provided in the literature while some were scaled linearly. The base geometric values used for scaling were obtained from Mundozah et al. [28]. To calculate the net forward velocity ($v_{p}$) the MRT for the experiment was used. Using MRT instead of powder feed rate is a more accurate for calculating $v_{p}$ as this calculation also accounts for the effects of hold-up of the powder within the barrels.However, in several cases the MRT was not reported and had to be estimated. Muddu et al. [44] have proposed a geometric correlation for the prediction of MRT but it has several fitting constants which does not enable scaling between different geometric scales. Thus, a statistical method was needed to be employed to complete the MRT values for the experimental data sets.

#### 3.2.2. Statistical Data Completion Techniques

The development of a granule regime depends upon both the process inputs as well as on the process outcomes. Some outcomes such as Torque and MRT do not have any first-principle based correlations for completion. These outputs need to be estimated using statistical methods. One of the most commonly used statistical method is linear regression [45]. In this study, a multivariate linear regression was used since the torque and MRT values for a TSG are dependent on several inputs instead of only a single input. A general representation of a multivariate regression is shown in Equation (10). The regression model was trained using the *sklearn* [46] package in Python [47]. The regression model used the existing data for torque and MRT for training.

(10) $$\begin{array}{c} Y=BX+\Xi \end{array}$$

where, Y is the response matrix of size n×p, X is the matrix containing all predictors with size of n×(q+1). *B* is a (q+1)×p matrix of fixed parameters, Ξ is the intercept matrix of size ntimesp. Here *n* represents the number of observations, *q* represents the number of inputs or predictors and *p* represents the number of responses or outputs. This model is often referred to as deterministic regression imputation. Such an imputation can add a bias to the predictions. In order to remove such biases, uncertainty can be added back to these models. Stochastic Regression Imputation [45] adds back normally distributed noise with a mean (μ) of zero and the variance ($\sigma^{2}$) equal to the standard error of predictions back to the regression estimates. Equation (11) represents a typical Stochastic Regression Imputation mathematically.

(11) $$\begin{array}{c} Y=BX+\Xi +N(\mu ,\sigma^{2}) \end{array}$$

Section 4.1 discusses the results of these techniques in more detail and the method chosen for each of these outputs.

### 3.3. Regime Map Formulation

The first step in the development of a granulation regime map is the formulation of the *x* and *y*-axis. These two axis are formulated such that they explain the key physical factors affecting the granulation rate mechanism studied in the regime map. For example, the batch granulation growth regime map developed by [17,24,48] was divided such that the *y*-axis explains the deformability of the granule and the *x*-axis explains the pore saturation, which is reflective of the amount of surface liquid present on the colliding granules. This section will elaborate on the formulation of each axes for the proposed regime map.

#### 3.3.1. X-axis

The granulation growth regime map being developed in this study is based on the common wet-granulation theory, that was explained earlier. In such regime maps, generally, the *x*-axis is formulated to account for the surface wetness of the colliding granules. Both the liquid to solid ratio and binder viscosity have a significant effect on the granule pore saturation and the strength of the liquid bridges formed Dhenge et al., 2012 and Mundozah et al. 2019. Hence, the product of these two parameters will be used to represent the surface wetness of the granules on the *x*-axis. To make this parameter a dimensionless parameter, relative viscosity (relative with respect to water) will be used instead of viscosity. The final formulation of the *x*-axis is x=L/Sratio×relativeviscosity.

#### 3.3.2. Y-axis

To incorporate Equation (8) or either of Equations (6) or (7) into regime map formulation, a large number of single granule experiments or testing on pellets is required. One of the aims of this study is to develop a granulation regime map that is more generalizable and widely applicable. However, at the same time, it is important to incorporate the complex deformation physics occurring during the granulation. For that reason, a simple model to capture the granule deformation will be developed in this study. This model was developed by leveraging the data from extensive single granule experiments performed by Fu et al. [34].

Fu et al. [34] experimentally studied the impact behavior of wet granules by projecting individual granules at a rigid target at predetermined speeds. The coefficient of restitution and maximum contact radius were measured for each run using high-speed cameras. Different granules were produced by varying the *L*/*S* ratio, binder viscosity, impact velocity, and primary particle size. Fu et al. [34] observed that a minimum impact velocity the impacts resulted in a zero coefficient of restitution and attributed it to type 1 coalescence (binder viscous dissipation). Post minimum impact velocity, the coefficient of restitution increases with impact velocity to a maximum value before becoming zero again. This second phase of zero coefficient of restitution is the result of type 2 coalescence and at high impact velocity, significant plastic deformation was observed which increased contact surface area between the particle and wall. Contact ratio, defined as the ratio between the post impact maximum strain and that of the radius of the granule, was also analyzed as part of this study. The contact ratio was observed to increase with impact velocity and liquid to solid ratio.

As the contact ratio represents the radial strain inside the deformed granule, it can be directly used as a measure of granule deformability. Hence, a multivariate regression model was developed to estimate the contact ratio as a function of the *L*/*S* ratio and impact velocity, as shown in Equation (12). The coefficient of determination ($R^{2}$), for this model, was observed to be about 0.98.

(12) $$\begin{array}{c} Contactratio=0.0735\times D_{p}+0.3486\times \sigma_{binder}\times L/S+0.1352\times vs.\times L/S \end{array}$$

Here, *v* is the impact velocity, $D_{p}$ represents the primary particle size, $\sigma_{binder}$ is the viscosity of the binder used and L/S is the binder liquid to solid ratio. Further, the *y*-axis of the regime map was defined in this study as the fraction of input energy that was dissipated during granule deformation. Commonly, the input energy to the TSG is defined as the specific mechanical energy (SME) [7], which is defined as the energy supplied to the TSG per unit gram, as shown in Equation (13).

(13) $$\begin{array}{c} SME=\frac{\tau \omega }{FR} \end{array}$$

Here, τ is the torque of the impeller, ω is the impeller speed in rad/s and FR is the feed rate. For this study, SME was modified as energy supplied to the powders per unit gram per unit second. Specifically, the denominator in Equation (6) was modified to hold up to obtain the actual estimate of the energy being applied on the powders inside the granulator (Equation (15)).

(14) $$\begin{array}{c} holdup\left(\phi \right)=FR\times MRT\times \frac{V_{free}}{V_{\max}} \end{array}$$

(15) $$\begin{array}{c} E_{s}=\;\frac{\tau \times \omega }{\phi } \end{array}$$

Here, MRT is the mean residence time (s), $V_{free}$ is the free volume available for the powders in the granulator, $V_{max}$ is the maximum volume available in the granulator and $E_{s}$ is the actual energy supplied by the granulator to the powders per unit time and weight. Finally, the *y*-axis was formulated as the product of $E_{s}$ and contact ratio, shown in Equations (12) and (15), respectively.

(16) $$\begin{array}{c} y-axis=E_{s}\times contact\;ratio \end{array}$$

The impact velocity (*v*), required for the contact ratio calculation as estimated as the square root of the energy supplied to the powders per unit time and mass ($E_{s}$).

## 4. Results and Discussion

### 4.1. Missing Data Completion

**Figure 1:**
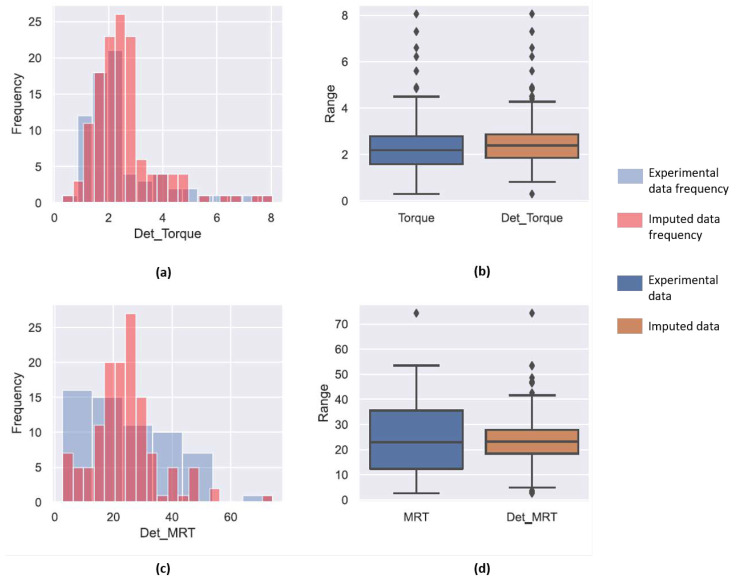
(**a**,**c**) compare the frequency of occurrence of each value of torque and MRT, respectively. The blue bar in the plot represent the experimental values while the red bars represent the values predicted by the regression model. A higher overlap indicates a regression model with good prediction accuracy. (**b**,**d**) are box plots comparing the median values as well as the range of experiments to the median and range obtained from the regression model for the torque and MRT, respectively. The diamond points represent points that lie beyond the statitical range.

**Figure 2:**
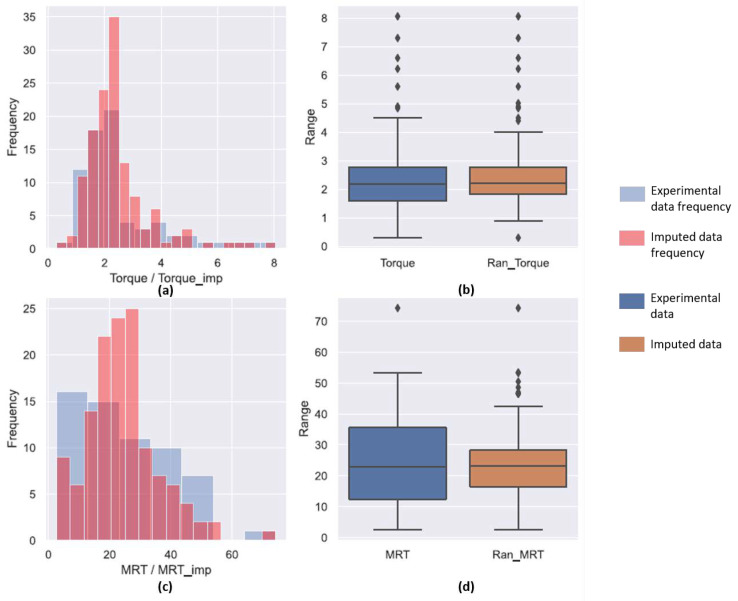
(**a**,**c**) compare the frequency of occurrence of each value of torque and MRT, respectively. The blue bar in the plot represent the experimental values while the red bars represent the values predicted by the stochastic regression model. (**b**,**d**) are box plots comparing the median values as well as the range of experiments to the median and range obtained from the stochastic regression model for the torque and MRT, respectively. A good stochastic regression model is one which can mimic the range of the experiments. The diamond points represent points that lie beyond the statitical range.

In the data set collected for analysis, it was observed that three variables that were studied had missing values for several experiments in the data set. Thus, in order to complete these values two statistical methods were employed in conjunction with physics-based correlations. The fill level correlation in Equation (1) requires the MRT for the process to calculated in order to estimate $F_{3}$. Thus, MRT was the first variable chosen to be completed. Since there were no physical correlations for estimating MRT, deterministic as well as stochastic regression imputation techniques were used. Both these techniques can to predict multiple dependent variables at once. Thus, the torque applied by the screws on the the granulating system and the MRT were estimated simultaneously. Figure 1 and Figure 2 contain the predictions for both the MRT and torque using these two data imputation techniques.

Figure 1a,c compare the already existing experimental data with the predictions from the deterministic regression imputation technique using a histogram plot. This histogram in blue represents the frequency of the torque and MRT experimental data, while the red overlay shows that frequency of the data set after it was completed using the deterministic regression imputation technique. The frequencies for the completed data set should align or be close to the distribution in the experimental data set, as most of the missing variables had process conditions within the range of previously performed experiments and no extrapolation was required. In the case of completion of torque values, it was observed that the distribution for both the experimental data and the completed data set was close to each other. This was also verified by the box plot present in Figure 1b. The blue box plot depicts the inter-quartile region (IQR) and the black line in the middle represents the median for the experimental data and the orange box represents the same for the completed data set. The upper and lower whiskers represent the maximum and minimum values for the distribution, respectively, while the diamond markers represent the outliers. Comparing the two box plots, it can be seen that the IQR for both these data sets are similar. The median for the completed data set is higher which can be attributed to the predictions of the incomplete variables. Thus, deterministic regression imputation proved to be a good technique the torque data.

Unlike the torque predictions, the predictions for the MRT of the incomplete experimental points was not representative of the given data. The distribution histogram in Figure 1c indicated that completed set had a higher frequencies for larger MRT and the distribution was narrow. This is also seen in the box plot in Figure 1d were the IQR for the predicted deterministic MRT value is narrow. The number of outliers for the completed data set were also higher. This meant that the deterministic completion did not account for variability in the data. Thus, stochastic regression imputation was used. The prediction of torque using stochastic regression imputation was similar to the one completed by the deterministic method due to low variance. However, this method was more effective in predicting the variations inherently present in the MRT data set. The stochastic regression imputed values for MRT also had lesser outliers and was more inclusive. Thus, this technique was chosen to complete the MRT data.

**Figure 3:**
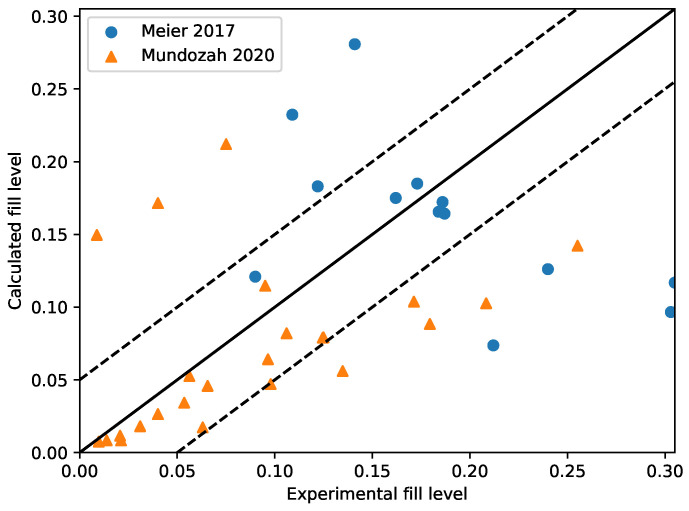
A parity plot comparing the predicted values of fill level with experimental values. The dotted lines represent confidence intervals of 0.05. The correlation has good prediction capability with 80% points lying between the 2 confidence intervals [28,36].

Equation (1) was used to complete the missing values for fill level within the twin screw granulator. Equation (5) used MRT values obtained from stochastic regression imputation. To check the effectiveness of the fill level correlation, predictions obtained were checked with two experimental data sets where fill level was available. Figure 3 shows a comparison between the predicted values obtained by using the first-principle based correlation and the experimental fill level reported by Meier et al. [36] and Mundozah et al. [28]. The average RMSE for the prediction was 0.073. Out of the 43 points predicted, 28 points are within a set confidence interval of 5% of fill level. The error in some cases seems to be high at 25% but a 7% change in fill level leading to a complete change in granule regime is not expected. Thus, the correlation used will provide a good understanding on the amount of material present inside the granulator at steady state.

**Figure 4:**
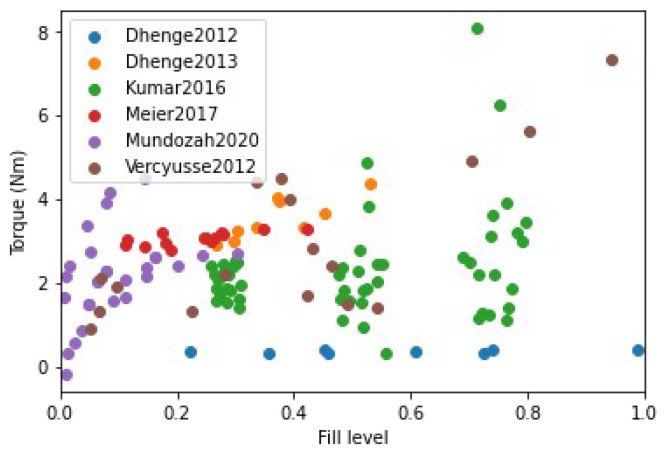
The variation of torque with fill level within the twin screw granulator. The points are color-coded at different *L*/*S* ratios. A general increase in torque values is observed with increase in fill level for a constant *L*/*S* ratio [5,15,19,28,35,36].

To verify that the data imputation methods used are effective, the final values of the torque predictions were also compared with previously published data sets. Mendez-Torrecillas et al. [42] through their experimental studies observed that torque for a system at constant *L*/*S* ratio increased with an increase in the fill level. A similar analysis was performed for the data completed for torque using the deterministic stochastic method and for the fill level using Equation (1). Figure 4 shows the plot of the torque values as a function of fill level. The data points are colored according to the *L*/*S* ratios used in the experiments and it can be observed that for a constant *L*/*S* ratio the torque values increase with an increase in the fill level inside the granulator. This is similar to the trends observed in previous literature and these predicted values can be used as good estimates for the variables when relevant data is not available.

### 4.2. Region Identification on the Granule Growth Regime Map

**Figure 5:**
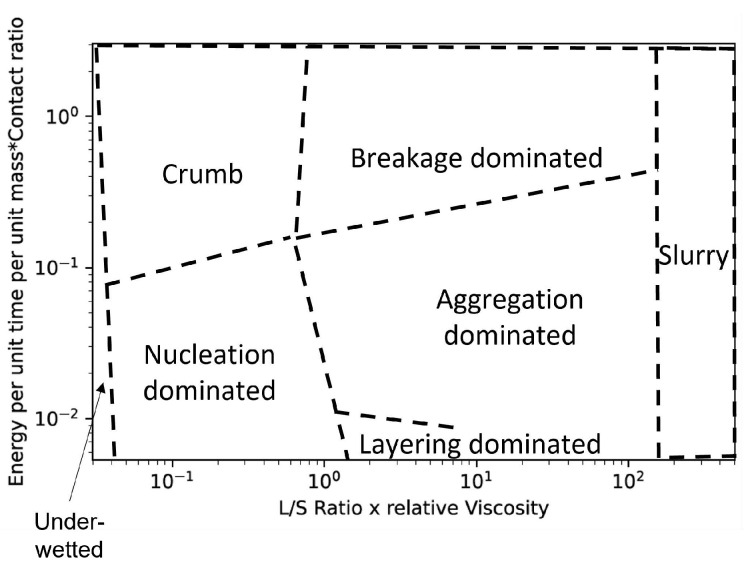
Proposed boundaries for the proposed regime map to differentiate between different growth granule regimes.

The position of a point on the 2-D map illustrated in Figure 5 of L/Sratio×relativeviscosity vs. $E_{s}\times contact\;ratio$ will determine the granulation mechanisms that the system has gone through along the length of the twin-screw granulator and the state of the granules obtained.

- At very low *x*-axis values of the proposed regime map, the binder content is very low, and no granules are formed. This region was named the “Under-wetted” region. Close to the right extreme of this *x*-axis value range, at high $E_{s}\times contact\;ratio$ there could be formation of crumb-like material due to Van der Waal and other such dry surface adhesion phenomena, this region falls under “Crumb”.
- At a slightly greater L/Sratio×relativeviscosity (estimated to be around 0.07 based on Iveson et al.’s work [24]) and at low $E_{s}\times contact\;ratio$, due to the lack of sufficient binder to form adequate nuclei the resulting granule size distribution is observed to have bi-modal distribution with a large percentage of fine particles and a relatively higher amount of loosely formed large particles. This is typical of nucleation dominant growth and hence, this region was named “Nucleation”.
- In this range of L/Sratio×relativeviscosity and at high $E_{s}\times contact\;ratio$ the loosely formed large particles undergo breakage and ultimately crumb-like material formation occurs. This region was also named “Crumb”.
- When the L/Sratio×relativeviscosity is further increased, sufficient binder becomes available to form more nuclei. Under such conditions, one of the following phenomena occurs depending on the $E_{s}\times contact\;ratio$ value.
  – At very low $E_{s}\;\times \;contact\;ratio$, due to a lack of sufficient energy for successful coalescence the granules will grow via layering mechanism. In previous work, Kotamarthy et al. [6] showed that the axial mixing and the collisions are affected by the energy supplied to the system, which is directly proportional to the mixing efficiency. Due to poor mixing and low shear conditions, these process and equipment settings can lead to the formation of bi-modal size distributions characterized by the formation of relatively smaller and weaker (higher porosity) granules. This is named “Type A/Layering dominant granulation”.
  – At higher $E_{s}\times contact\;ratio$, there is sufficient energy to squeeze the binder liquid to the surface of the granules leading to further coalescence and growth (Type II coalescence). Due to enhanced mixing and promotion of coalescence, granulation under these conditions will result in a relatively uni-modal particle size distribution. Moreover, due to extensive coalescence, the granules formed under these conditions will be highly dense and relatively large. This region was named “Type B/coalescence driven granulation”.
- When the L/Sratio×relativeviscosity is increased further there is sufficient binder liquid present around the initial granules formed to promote coalescence type growth (Type I coalescence) irrespective of the energy provided. Such settings will lead to the formation of very large granules. This region will still fall under “Type B/coalescence driven granulation”.
- Very high L/Sratio×relativeviscosity will result in the formation of paste-like material. This region was named “Slurry”.

### 4.3. Effect of Twin-Screw Granulation Parameters

#### 4.3.1. Effect of Screw Speed

As the screw speed increases the energy supplied per unit time per unit mass to the powders increases ($E_{s}$). An increase in screw speed leads to an increase in axial velocity of particles which ultimately decreases the mean residence time of particles inside the system [6,49,50]. In this study, impact velocity is calculated as the square root of the energy supplied per unit time per unit mass ($E_{s}$). Moreover, studies have shown that an increase in hold up or fill level will increase the resistance to the rotation of the impeller, thereby increasing the torque applied by the machine [35,36,42]. From Equation (14) it can be seen that a decrease in MRT decreases the holdup and fill level. Since an increase in screw speed leads to a decrease in MRT, it ultimately leads to a decrease in Torque. Vercruysse et al. [35] and Mendez et al. [42] have shown that the influence of screw speed on Torque drastically decreases at higher screw speeds. This was attributed to an insignificant change in fill level caused by a change in screw speed at higher screw speeds. Therefore, an increase in screw speed leads to an increase in the contact ratio and a decrease in MRT and Torque.

In several scenarios, an increase in screw speed will lead to a positive effect in the *y*-axis of the proposed regime map, especially because of its direct positive effect on granule deformability in terms of screw speed and contact ratio, compared to the indirect negative effect on Torque. Hence, an increase in screw speed leads to an increase in the *y*-axis of the proposed regime, which leads to an increase in granule deformation. Therefore, increasing the impeller speed will shift a systems’ behavior to the top of the proposed growth regime map, that is, from nucleation to crumb; from induction to steady; from steady to “breakage-dominated” steady growth.

#### 4.3.2. Effect of Feed Rate

Feed rate has competing effects on holdup and fill level, and as shown in Equation (7) it has a direct positive effect on hold up. However, it also affects MRT such that an increase in feed rate leads to a decrease in MRT [21,44,50]. This is attributed to the increase in powder throughput force with an increase in feed rate, and this decreases the occurrence of back mixing and eventually leads to an increase in axial velocity and decrease in MRT. Mundozah et al. [51] and Meier et al. [36] showed that even though there is a decrease in MRT with an increase in feed rate, the increase in the holdup and fill level prevailed. This can be attributed to the nature of the effect feed rate has on MRT and holdup, which are also dependent on other factors such as screw speed and screw configuration [50], which decreases the influence of feed rate on MRT. Hence an increase in feed rate has a net positive effect on holdup, and an increase in hold up leads to an increase in the torque of the system. Studies have shown that a large increase in feed rate only increases the Torque by a few percentage points [19,52,53], and this has been attributed to an insignificant increase in resistance to the powder flow due to an increase in powder throughput force and a decrease in the occurrence of back mixing. Since feed rate has a direct positive effect on hold up and only has a small indirect positive effect on Torque, the energy per unit gram per unit time decreases with an increase in feed rate.

An increase in feed rate leads to a decrease in energy supplied to the powder per unit time per unit mass ($E_{s}$). This will in turn cause a decrease in the impact velocity and therefore, a decrease in the contact ratio. All of these factors contribute to a decrease in granule deformation. Therefore, an increase in the feed rate shifts the system’s behavior to the bottom of the regime map, that is, from crumb to nucleation; from steady to induction; from breakage-dominated steady growth to steady growth.

#### 4.3.3. Effect of Liquid to Solid Ratio

The liquid to solid ratio (*L*/*S*) has a direct positive effect on the *x*-axis. An increase in the liquid to solid ratio increases the pore saturation of the granules and eventually leads to an increase in available liquid on the surface of the colliding granules [17,24]. This increase in surface wetness will ultimately lead to an increase in granule growth. The *L*/*S* ratio also affects the *y*-axis of the proposed regime map. An increase in the *L*/*S* ratio increases the contact ratio between colliding granules, as shown in Equation (12). Apart from this the *L*/*S* ratio also affects MRT and torque supplied to the granules, which in turn affect the energy supplied per unit mass per unit time. Dhenge et al. [5,21] have reported that an increase in the *L*/*S* ratio leads to an increase in resistance to the flow due to an increase in viscous forces on the powder particles. This leads to an increase in both MRT and Torque. Kumar et al. [52] reported that the *L*/*S* ratio had a significant interaction effect with feed rate and screw speed on the torque. They found that at a high fill level an increase in the *L*/*S* ratio resulted in a reduction of Torque and the opposite effect was observed at a low fill level. This can be attributed to the fact that at a high fill level (torque is already high due to the presence of a large amount of material in the barrel), an increase in the *L*/*S* ratio leads to the formation of a greater number of granules, thereby increasing the local flowability and decreasing the resistance. This will eventually lead to a decrease in Torque, whereas at a low fill level (resistance to powder flow is low and hence the torque is low) an increase in the *L*/*S* ratio increases the viscous forces on the powder particles increasing the resistance to flow and torque supplied by the equipment. Kumar et al. [52] also reported that the *L*/*S* ratio did not have a dominant influence on the MRT, and it had a positive impact on the MRT only at a high fill level.

Since the effect of the *L*/*S* ratio on torque and MRT is highly dependent on other process parameters, its influence on the contact ratio will be more significant compared to the other parameters. Hence an increase in the *L*/*S* ratio leads to an increase in both the *x*-axis and *y*-axis of a regime map. Therefore, an increase in the *L*/*S* ratio will move the system to the top right corner of the regime map, i.e., from the “nucleation” to the “steady growth”, from the “steady growth” to the “breakage dominated steady growth”, from the “steady growth” to the “slurry”, from the “breakage dominated steady growth” to the “slurry”.

#### 4.3.4. Effect of Binder Viscosity

The binder viscosity also has a direct positive effect on the *x*-axis of the proposed regime map. In twin-screw granulation, the binder liquid is generally added by a dripping mechanism due to the design of the equipment. In such as system, the nucleation mechanism is controlled by its kinetic parameters such as drop penetration time and the spreading of the liquid droplet over the solid powder [51,54,55]. The drop penetration time decreases with an increase in binder viscosity, the spreading of the drop on the powder surface also decreases [19,51]. This leads to an increase in the available surface liquid on the granule, leading to the formation of strong and dense granules [19].

Binder viscosity also affects the *y*-axis of the proposed regime map, and an increase in the viscous nature of the binder (thereby the powder-binder mixture) leads to an increase in the MRT and the holdup in general [5,7], but the significance of this effect on torque is dependent on the screw configuration and mixing efficiency in the granulator. In the conveying zone, the decrease in the rate of nucleation kinetics due to an increase in binder viscosity leads to granule formation via layering mechanism [20]. In the presence of kneading elements (minimum shear) the loosely formed nuclei and granules (formed via layering) are broken and the liquid is redistributed, enhancing the liquid–solid mixing [39,51,56,57,58]. This further increases the viscous nature of the entire powder bed, leading to a significant increase in torque and granule growth. Essentially there is a granule growth mechanism shift from layering dominated to coalescence dominated and this leads to an increase in the significance of binder viscosity on torque. The above-mentioned theory combined with mechanical dispersion offered by the screw configuration affects the torque and granulation mechanisms occurring along the length of the screws in a twin-screw granulator. Hence an increase in binder viscosity leads to an increase in the energy supplied by the system per unit time per unit mass, especially in the presence of the kneading elements. Moreover, binder viscosity has a direct positive effect on the contact ratio (Equation (12)).

Similar to the *L*/*S* ratio case, due to the nature of effects, an increase in binder viscosity will ultimately increase the *y*-axis value of the proposed regime map. Therefore, an increase in the binder viscosity will move the system to the top right corner of the regime map, i.e., from the “nucleation” to the “steady growth”, from the “steady growth” to the “breakage dominated steady growth”, from the “steady growth” to the slurry”, from the “breakage dominated steady growth” to the “slurry”.

#### 4.3.5. Effect of Kneading Elements

An increase in the number of kneading of elements decreases the free volume inside the barrel ($V_{free}$) [28,51], and this leads to an increase in blockage of powders in the zones surrounding the kneading block in the barrel. This increase in blockage will eventually lead to an increase in MRT and holdup [7,28,37,52]. The increase in holdup due to an increase in kneading elements leads to an increase in barrel fill level which ultimately leads to an increase in system torque. Kumar et al. [52] reported that kneading elements are the principal factor affecting the system torque values, whereas the influence of kneading elements on MRT is dependent on powder feed rate and screw speed. Taking all these effects into account the net effect of kneading elements on the energy supplied per unit mass per unit time ($E_{s}$)to the powders, is a positive one. As mentioned earlier, $E_{s}$ can be translated as the stress applied on the powders inside the TSG and many studies have reported that the kneading elements provide the shear required for granulation in a TSG [7,15,39,42,59,60,61,62]. Since the energy supplied per unit mass per unit time ($E_{s}$ ) increases with the increase in the number of kneading elements, the impact velocity will also increase. This will, in turn, lead to an increase in contact ratio and deformability of the granule. Therefore, with an increase in the number of kneading elements, the *y*-axis of the proposed growth regime map will also increase. This will shift the systems’ behavior to the top of the proposed growth regime map, that is, from nucleation to crumb; from induction to steady; from steady to “breakage-dominated” steady growth.

#### 4.3.6. Effect of Stagger Angle

Similar to the effect of kneading elements, with the increase in stagger angle the free volume inside the barrel decreases, ultimately leading to an increase in MRT and hold up. It has been reported in the literature that the effect of stagger angle on MRT is insignificant at low stagger angle (30deg or 60deg) and high screw speed or high throughput values [7,22,37,50]. The stagger angle significantly affects the conveying and mixing capacities of the kneading zone [63,64,65,66]. It has been shown that at low forward stagger angles the conveying capacity is very high compared to large forward and reverse angles [20]. Essentially the pseudo helix angle created by the staggering of kneading elements at different angles dictates the extent of back mixing and length of the back mixing zone [20]. Therefore, at a low stagger angle setting, when the conveying capacity of the kneading zone is high, the screw speed, throughput, and the number of kneading elements have a more dominant effect on MRT and thereby on torque, whereas at high forward angles and reverse angles, the back mixing within the twin-screw granulator barrel is significantly higher which in turn leads to a significant increase in MRT and holdup. It was shown in the literature that after a certain critical fill level the torque provided by the system increases exponentially with an increase in holdup/fill level [42]. It could be said that those levels of fill level can be achieved with high forward and reverse stagger angles combined with a medium to a high number of kneading elements.

Though the stagger angle might have an insignificant effect on the energy per unit mass per unit time ($E_{s}$) at low forward angles, at high stagger angles it has a net positive effect on $E_{s}$. This in turn leads to an increase in impact velocity and contact ratio. Hence with an increase in the stagger angle, the *y*-axis of the proposed growth regime map will also increase. This will shift the systems’ behavior to the top of the proposed growth regime map, that is, from nucleation to crumb; from induction to steady; from steady to “breakage-dominated” steady growth.

#### 4.3.7. Effect of Primary Powder Properties

It can be seen from Equation (12) that primary particle has a direct positive effect on contact ratio, and this was due to the decrease in Young’s modulus of the granule with an increase in the particle size [34]. Therefore an increase in particle size will move the system to the top of the proposed regime map. An increase in bulk density of primary powders leads to an increase in the hold up of the system [67], due to an increase in the packing fraction of the powders within the barrel. However, the overall effect of the increase in bulk density depends on its interaction with process and screw parameters.

**Figure 6:**
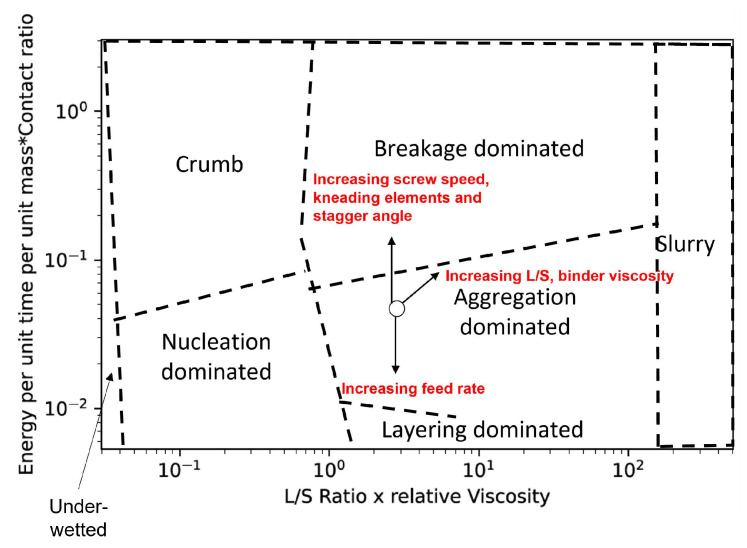
The influence of the process and the screw parameters on the regime map.

It is difficult to isolate the effect of primary powder particles characteristics such as particle shape, roughness, density, etc. on granulation behavior. Therefore these were not studied here, but it is expected that the effect of these powder properties will implicitly be accounted for by their effect on properties such as MRT, torque, etc. In order to incorporate the effects of these primary powders better, further detailed experiments need to be carried out. The effects of all the important process and screw parameters are summarized in Table 3 and their influence on the regime map is plotted in Figure 6.

### 4.4. Validation of Developed Regime Map

**Figure 7:**
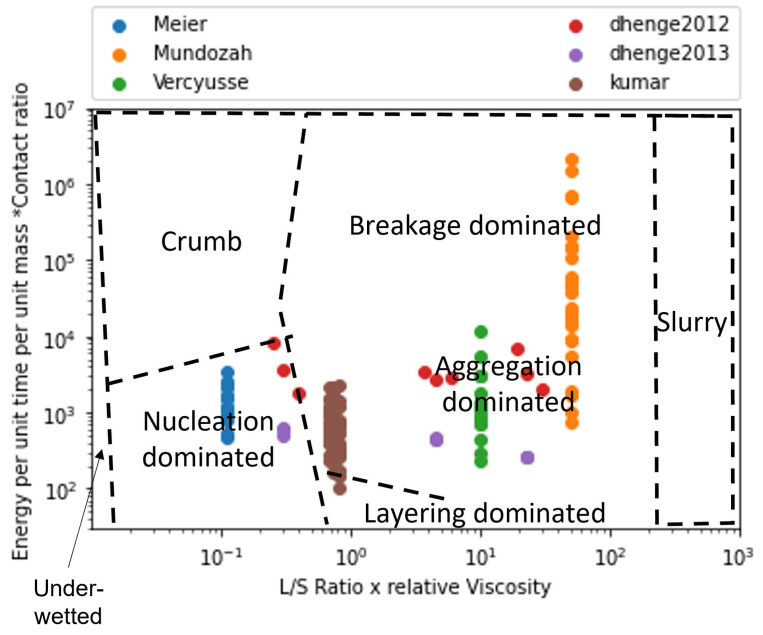
The regime map developed with proposed boundaries and each point colored according to the experimental study used in development. The regimes described by the boundaries in this map can explain the experimentally observed regimes with good accuracy [5,15,19,28,35,36].

The formulated *x* and *y*-axis were calculated for all the 132 collected data points from the 6 experimental studies and were plotted on the regime map as shown in Figure 7. Figure 7 has markers colored according to the experimental study they were obtained. A large variation can be observed when all the experimental points are plotted on the regime map as different studies used different experimental design of experiments (DoE). Besides, several of these studies had different formulation parameters. It can be observed that many of the of the experimentl data points within a particular study were clustered together (Figure 7).This could be expected because each study only analyzed a small variation of the process parameters in their DoE’s. This clustering would also mean that all the experiments within each of the experimental study had similar granule growth kinetics. Only two studies analyzed and reported varying granule growth kinetics in their study [5,19]. This was achieved by varying the binder viscosity during their experiments which led to large variation across the *x*-axis.

**Figure 8:**
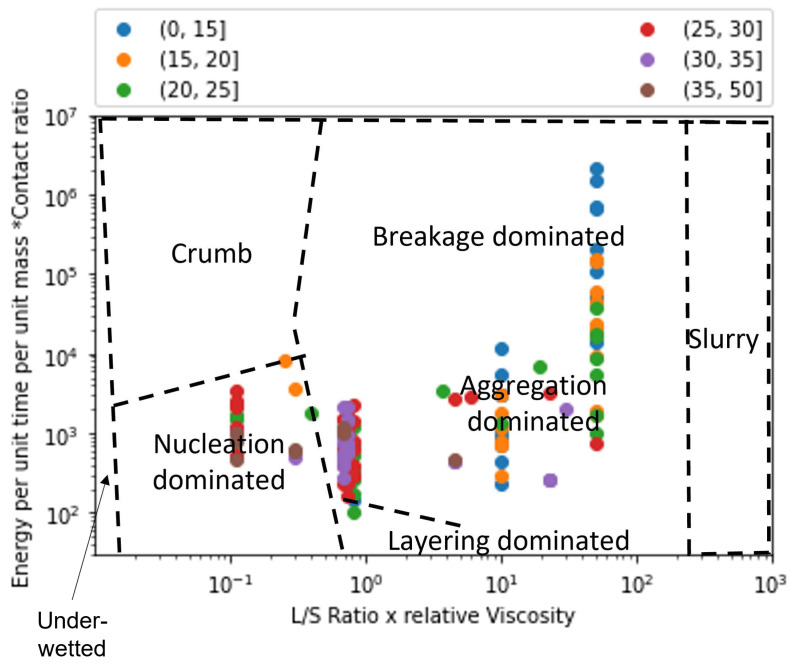
The regime map developed with proposed boundaries and each point colored according to the extent of granule formation. Extent of granule formation is defined as the ratio of the final median diameter of the obtained granules to the initial median diameter of the powder blend.

Extent of granule formation is defined as the ratio of the final median diameter of the granules ($d_{50}$) to the initial median diameter of the powder blend. This measure is a direct reflection on the growth of the granule and can aid in understanding the type of granule growth that might have occurred during the twin screw granulation process. In Figure 8, the experimental data points are plotted on the proposed regime map with boundaries determined in Section 4.2. The experimental points are colored based on the extent of granulation with ranges varying from 0 to 50. In order to figure out the granule growth regime occurring in each of these experimental data set, Figure 7 and Figure 8 were analyzed together. Meier et al. [36] employed an *L*/*S* ratio of 0.11 with 12 kneading elements set at 60 degree stagger angle while varying the screw speed and feed rate between 138 & 562 rpm, and 1.134 & 8.554 kg/h, respectively. They used Hydrochlorothiazide as their input material which is an insoluble hydrophobic powder [68]. It has been shown in the literature that a hydrophobic powder requires the usage of a large *L*/*S* ratio for robust granule growth [57]. Moreover, Mundozah et al. [69] showed that a stagger angle of 60 degree was unhelpful to promote liquid solid mixing and granule growth for such formulations. Hence, under such conditions small extent of growth can be expected, as seen in the case of Meier et al.’s study [36] (Figure 8).This confirms our finding that the conditions employed by Meier et al. [36] leads to a nucleation and crumb type of growth in the TSG (Figure 7).

**Figure 9:**
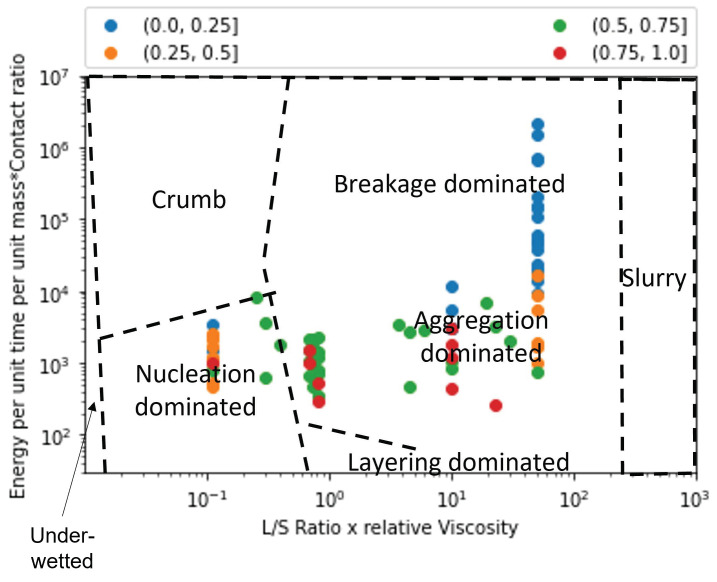
The regime map developed with proposed boundaries and each point colored according fill level within the barrel.

Vercruysse et al. [35] used a mixture of Theophylline and Lactose as their input material, which are both hydrophilic. Robust granulation of hydrophilic components can be achieved in a TSG at very low *L*/*S* ratio [70,71,72], especially in the presence of kneading elements [69]. Moreover, Vercruysse et al. [35] also used PVP as a binder which increased the relative density of the liquid binder close to 100, while keeping the *L*/*S* ratio constant at 0.09, Vercruysse et al. [35] varied the number of kneading elements, stagger angle, screw speed and feed rate between 2 & 12, 30 & 90, 600 & 950, and 10 & 25, respectively. Under these settings they achieved an extent of granulation as high as 25. Hence, it can be safely assumed that for this study the granule growth in the TSG occurred in the aggregation dominant region (Figure 8 and Figure 9).

Kumar et al.’s [15] study used Lactose as the input material for their study. The number of kneading elements (6 & 12), *L*/*S* ratio (0.08,0.09,0.1), screw speed (500,700,900), feed rate (10,17.5,25 kg/h) and binder viscosity (6.9,8.3,10.3 mPa.s) were varied, while keeping stagger angle was kept constant at 60 degrees. Lactose being soluble and hydrophilic, under these conditions produced granules with a large extent of granulation. Forty three of the experimental runs produced granules with an extent of granulation grater than 25. of the remaining 11 runs 8 runs produced an extent of granulation between 20 and 25. This indicates that the majority of the granulation occurred via. aggregation dominated growth. Kumar et al. [15] observed that for one of the run the resultant barrel torque was very low (0.3 Nm), consequently this experimental run produced an extent of granulation as low as 10. Hence, it can be anticipated that this run could have followed a more layering driven growth due to lack of shear in the TSG, as shown in Figure 7 and Figure 8.

Dhenge et al. [5,19] carried out two studies to develop a regime map for the TSG. In the first study [5], *L*/*S* ratio and binder viscosity were varied by employing a screw configuration with two kneading zones arranged at a stagger angle of 60 degree. For this study, authors kept the screw speed and feed rate constant at 400 rpm and 3.5 kg/h, respectively. A mixture of Lactose, Avicel and crosscarmellose sodium, with Lactose as the major component (80%w/w), was used as the input material. The extent of granulation for the granules obtained in this study was greater than 17.5 for all the runs, with majority of the runs having an extent of granulation greater than 20 and the maximum reaching close to 35. This study also reported micro-CT scans of the granules, based on these and the extent of granulation runs it can be concluded that at the lowest *L*/*S* and lowest viscosity values the granules would be in the cusp of crumb, nucleation and aggregation-dominated growth region and the remaining experimental runs would fall in the aggregation-dominated growth region. This is due to both the sufficient amount of shear and liquid available in the system leading to a mixture of Type 1 and Type 2 coalescence. These findings match the conclusions of Dhenge et al. [5,19], where they marked the regions of interest as “crumb”, “granules” and “bigger granules”.

The second study carried out by Dhenge et al. [19], employed the same formulation but used an only conveying element screw configuration. Dhenge et al. [19] varied feed rate and binder viscosity, while keeping the screw speed and *L*/*S* ratio fixed at 400 rpm and 0.3 and concluded that due to relatively low shear conditions of the only conveying element configuration, all the experiments followed a “nuclei” formation regime. However, the analysis of extent of granulation showed that for all the experiments the extent of granulation lied in the range of 25 to 35. This shows a large growth of granules which is uncharacteristic of nucleation-dominated growth. Furthermore, by analyzing the micro-CT scans and granule strength’s, reported by Dhenge et al. [19], it was found that all the runs, barring the lowest binder viscosity and feed rate condition, had dense cores and relatively high strengths (0.6–1 MPa). Based on these numbers it can be safely estimated that all these runs fall under the aggregation-dominated region and the lowest binder viscosity and lowest feed rate run falls in the cusp of nucleation, crumb and aggregation-dominated regime.

Mundozah et al. [28] studied the effect of barrel fill level on the extent of granulation. The barrel fill level was varied by varying the screw configuration, screw speed and powder feed rate. Mundozah et al. [28] showed that PMMA, a material with larger initial particle size and higher sphericity, achieved lower extent of granulation but showed a positive proportionality with the fill level. On the contrary for a material such as Lactose, a brittle material with smaller initial particle size, an overall greater extent of granulation was obtained but the extent of granulation started to decrease after a certain fill level. This was attributed to the dominant breakage mechanism obtained at higher fill level due to increased compressive stress on the granules. This observation was confirmed in the regime map analysis, it was observed that with increase in the *y*-axis value a general decrease in the extent of granulation was observed (Figure 8). This was a consequence of the regime shift from an aggregation-dominated growth to that of a breakage-dominated growth, as seen in Figure 7 and Figure 8. As expected, no experimental studies operated their experiments in the slurry or under-wetted regime.

### 4.5. Analysis of The-Breakage-Dominant Growth

It has been reported in the literature that breakage is a dominant mechanism in twin-screw granulation because of the restrictive volume available and high shear environment in the TSG barrel [14,73,74]. Breakage of granules promotes liquid–solid redistribution and subsequently aids in growth of the granule [12,59]. To understand and incorporate breakage better into the regime map further analysis of breakage-dominant growth was performed by investigating the contact ratios. The 132 experimental runs investigated in this study were first characterized on the basis of contact ratio. These segregated data sets were further characterized on the basis of extent of granulation. An extent of granulation of 20 was chosen as the defining number to analyze breakage as majority of these runs had an initial particle size of 50 micrometers. An extent of granulation of 20 would then result in the granules having a $d_{50}$ of 1000 micrometers. It was reported in the literature that the granule growth caused by a breakage-dominant mechanism in the TSG would result in granules having a $d_{50}$ of lower than 1000 micrometer or having an extent of granulation lower than 20 [12,28]. It was found out that out of the 132 experimental data points investigated in this study, 97 experiments resulted in granules having contact ratio <1. Of these 97 runs, 34 runs had an extent of granulation >20. Most of these runs fell under the nucleation and crumb regime, indicating poor granulation due to lack of sufficient binder liquid. The granules formed under such conditions are expected to have low deformability values as indicated by their contact ratios. Of the 35 runs that had a contact ratio of >1, only 12 runs had an extent of granulation greater than 20 (<35%).

**Figure 10:**
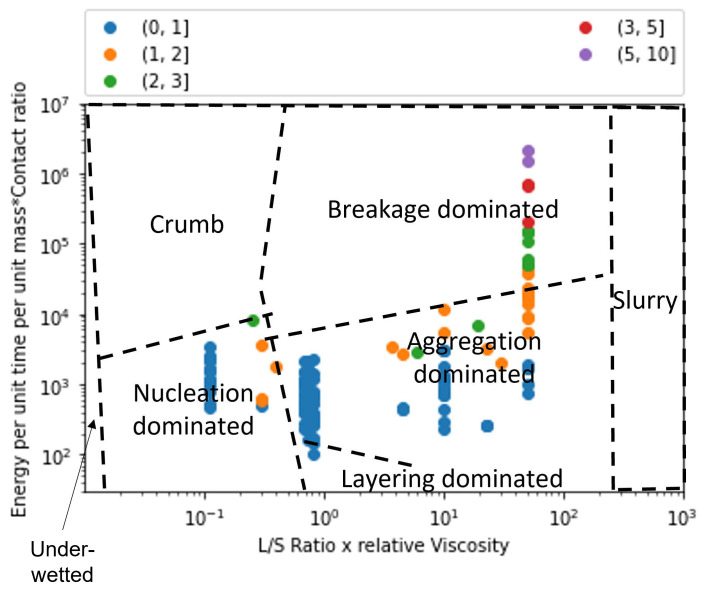
The regime map developed with proposed boundaries and each point colored according contact ratio of the granules.

Moreover, it was observed from Mundoazah et al.’s study [28] that an extent of granulation of >20 was observed in few cases even though the granules had undergone breakage. This analysis shows that contact ratio of greater than 1 could be a good indication of the breakage-dominant regime map, albeit sufficient *L*/*S* ratio and binder viscosity condition has to be met. From Figure 10 it can be observed that a contact ratio of greater than 2 was only achieved at high *y*-axis values and this validates our earlier theory and demarcation of the breakage-dominated growth regime in the regime-map. Keeping in mind that an increase in *L*/*S* ratio leads to granules with greater deformability, a slight positive slope is given to demarcation line between aggregation-dominated and breakage dominated regimes. Since majority of the experimental data points have used soluble, hydrophilic formulation, the precise boundaries for the transition of the granule growth regime needs further investigation. Furthermore, the investigation of factors other than *L*/*S* ratio and velocity on the contact ratio and consequently on the regime map requires further study.

## 5. Conclusions

Twin screw granulation is a versatile wet granulation technique that includes process parameters, geometrical parameters, and material properties as control parameters. Though this increases the applicability of this granulation technique, the interplay between these effects makes this process very complicated. Hence it is very important to understand and predict the granule growth apriori. This study addresses the problem via the development of a modified regime map. The developed regime map can be used to predict the type of growth a granule undergoes, and the resultant nature of the granules based on the process, material and design conditions of the TSG. There are two major advantages of this regime map; firstly, this regime map was accurately validated (90% of the experimental points were accurately estimated for their granulation growth regimes) against a vast variety of historical data present in the literature making it a widely applicable technique. Secondly, the applicability of this technique was simplified due to the development of mechanistically relevant reduced order parameters as the axes, which can be easily estimated. Furthermore, the incorporation of breakage-dominant growth in the regime map helped improve the understanding of granule growth behavior in twin-screw granulation. Data completion techniques were used wherever necessary, to complete important missing outputs from the chosen data sets. The data obtained from these techniques were validated according to the trends present in the literature. These techniques showed good prediction capabilities and can be adapted to a range of data sets. Several previously published experiments utilized Lactose Monohydrate as their input material, therefore, to improve the boundary and regime identification more data using different materials needs to studied using the developed regime map.

## Figures and Tables

**Table 1:** Experimental data collected for the development of the modified TSG regime map.

No.	Author	Granulator Size (mm)	Process Parameters Varied	Material Properties Varied	No. of Screw Configurations Used	No. of Experiments Performed
1	Dhenge et al., 2012 [5]	16	*L*/*S* Ratio	Binder viscosity	1	9
2	Vercruysse et al., 2012 [35]	25	Flow Rate			
			RPM	–	6	18
			Temperature			
3	Dhenge et al., 2013 [19]	16	Flow Rate	Binder viscosity	1	9
4	Meier et al., 2013 [36]	16	Flow Rate RPM	–	1	14
5	Kumar et al., 2016 [15]	25	Flow Rate			
			RPM	Binder viscosity	2	54
			*L*/*S* Ratio			
6	Mundozah et al., 2020 [28]	16	RPM	Bulk Solid	3	28
			**Total**			**132**

**Table 2:** Inputs and outputs for the development of the regime map.

Input Parameters			Outputs Parameters
Geometry	Process	Material	
Number of CE and KE	*L*/*S* ratio	Initial PSD	Granule size distribution (GSD)
Staggering angle of KE	Screw Speed (RPM)	Viscosity of binder	Torque
L/D ratio	Feed rate	% API in powder	% fill of barrel
Granulator diameter	Temperature		Mean residence time
Liquid addition position			

**Table 3:** Influence of the process and screw parameters on the important *x*-axis and *y*-axis parameters. (’D’ indicates direct effect, ’I’ indicates indirect effect, ’+’ indicates increasing trend and ’-’ indicates decreasing trend.)

		Process Parameters				Binder Liquid Paramters				Screw Parameters			
		Screw Speed		Feed Rate		*L*/*S* Ratio		Binder Viscosity		Kneading Elements		Stagger Angle	
	Parameters	D	I	D	I	D	I	D	I	D	I	D	I
*x*-axis	*L*/*S* Ratio					+							
	Binder Viscosity							+					
*y*-axis	Screw Speed	+											
	Torque		-		+		+		+		+		+
	Feed Rate			+									
	MRT		-		+		+		+		+		+
	$V_{Free}/V_{max}$							+		-		-	
	Contact Ratio		+		-		+		+		+		+

## Data Availability

Data can be made available on request.
